# Shifts in energy allocation and reproduction in response to temperature in a small precocial mammal

**DOI:** 10.1186/s40850-023-00185-6

**Published:** 2023-10-18

**Authors:** Fritz Trillmich, Anja Guenther

**Affiliations:** 1 Department of Animal Behaviour, University Bielefeld, Bielefeld, Germany; 2 Max-Planck Institute for Evolutionary Biology, RG Behavioural Ecology of Individual Differences, Plön, Germany

**Keywords:** Guinea pig, Fat storage, Growth, Lean body mass, Weaning Thermoregulation

## Abstract

**Background:**

Species adjust to changes in temperature and the accompanying reduction in resource availability during the annual cycle by shifts in energy allocation. As it gets colder, the priority of energy allocation to maintenance increases and reproduction is reduced or abandoned.

**Results:**

We studied whether and how young female guinea pigs (*Cavia porcellus*) adjust even under ad libitum food conditions growth, storage of fat reserves and reproduction when kept at 5 °C versus 15 °C, and how offspring born into these conditions compensate during development to independence. Reproducing females grew less in the cold. Their lower weight resulted largely from less fat storage whereas growth in fat free mass was about the same for both groups. The increased need for thermoregulation diminished fat storage most likely due to the development of more brown fat tissue. Reproductive activity did not differ between groups in terms of litter frequency, mass and size. However, females in 5 °C weaned pups later (around day 25) than females in 15 °C (around day 21). Later weaning did not make up for the higher energy expenditure of pups in cold conditions leading to slower growth and less fat storage. Female pups born into the cold matured later than those born in 15 °C. Investment in reproduction continued but allocation to individual pups declined.

**Conclusions:**

In more thermally demanding conditions female guinea pigs - even under ad libitum food abundance - transfer the higher costs of maintenance and reproduction largely to offspring.

## Background

Animals acquire energy from food through metabolic processes and allocate the available energy to functions such as survival (maintenance), growth, and reproduction. When the demand of energy for survival increases due to reduced food abundance or harsher environmental conditions animals may shift the allocation of available energy among these different functions. A typical example is the cessation of reproduction during winter time in many seasonally breeding animals [1]. If the change in environmental conditions is predictable, animals may prepare for the expected challenges: Birds massively store fat reserves before migration [2] and hibernating mammals prepare for wintering by increased food intake and high priority of fat storage [3]. If changes in the environment become less predictable or completely unpredictable and if animals can partly compensate by increasing food intake [4] it becomes harder to say how we expect an animal to change its energy allocation. For example, adult female house mice reproduce even in subfreezing temperatures as long as food supply is not restricted [5].

Animals with a long reproductive period may well experience shifts in resource availability and may need to allocate resources increasingly to self-maintenance. If they keep reproducing this raises the question how they react to such variability in resource abundance. Many rodents reproduce over much of the annual cycle and often face fluctuations in resource abundance due to changing weather conditions. This may force them to shift resource allocation and hence predicts a certain plasticity in allocation pattern [6].

This applies also to the cavy (*Cavia aperea*; [7]) a closely related wild species of the domesticated guinea pig (*Cavia porcellus*). In its natural habitat in Uruguay, *Cavia magna*, a closely related species, reproduces from spring to late autumn (and rarely even in winter) and encounters highly variable conditions during this period with respect to both, temperature and food abundance [8]. This variability influences reproduction in the cavy and leads to less intense reproduction in autumn and winter [7]. Under ad libitum food conditions, cavies show similar littering probabilities and intervals between litters when the temperature is reduced from 20 °C to 5 °C and when photoperiods indicate long (14:10 L:D) or short (9:15 L:D) days [9]. However, under naturally varying photoperiods and temperatures, pups born in unfavourable seasons such as winter and spring, have increased mortality rates and decreased growth rates [10]. In addition, mothers giving birth in unfavourable seasons show reduced reproductive effort indicated by smaller litter sizes and reduced birth rates [7]) indicating that they adjust their allocation strategy between reproduction and self-maintenance to prevailing environmental conditions. Diminished reproductive effort in unfavourable seasons is mediated physiologically by reduced fertilisation rates, less intense oestrus and fewer oocytes released during each reproductive cycle [7, 11].

In this context, we wanted to investigate in detail how a continuous difference in temperature (5 °C versus 15 °C) influences allocation decisions between reproductive effort and self-maintenance in guinea pig females. We chose the domestic form because we had previously established a method that permitted to measure the body fat content of guinea pigs [12] which allowed us to estimate shifts in allocation among growth, storage and reproduction.

Specifically we asked: (1) When females under ad libitum food conditions are forced to expend more on their own maintenance due to low temperature, how does this influence their allocation of resources to the functions of growth, build up of reserves and reproduction. (2) If offspring start life with lower body condition (i.e. lower birth mass) under more energetically demanding conditions, how do they respond to the situation with respect to allocation to growth, storage and development to independence?

## Methods

### Animals and procedures

For our experiments, guinea pigs of the outbred domestic stock kept at the Institute of Animal Behaviour, Bielefeld, were bred under room temperature (21 ± 2.5 °C). Female pups were kept together with their mother until day 30. At 30 days of age the experimental females were transferred into temperature-controlled rooms. One group was kept at 15 °C, the other at 5 °C. For each temperature condition, six groups of two females together with one male each were established. These groups were kept together for as long as the experiment lasted. The male was older than the females and clearly mature. He had been placed into the room two weeks previously. A group of two females with one male was kept in an enclosure of 0.8 m² floor area covered with wood chips for bedding and shelters. The animals received guinea pig pellet food (Höveler, Langenfeld, Germany) and water ad libitum. Guinea pigs cannot synthesize vitamin C, therefore, the drinking water was supplemented with ascorbic acid once a week (Roth, Karlsruhe, approximately 1 g/l). Additionally, the animals received hay and fresh food (carrots, apples or beets) almost daily. The rooms had only artificial light for 14 h per day (Philips TLD 58 W/965; 253 lx; digital luxmeter, Beha, Glottertal, Germany).

In an additional small experiment, we kept adult females with males in 5 and 20 °C using the same grouping and maintenance conditions as described above. Pups born to these females were left with the adults until day 40 when the males were removed. We determined whether female pups born into these conditions (13 females in 5 °C and 10 in 20 °C) conceived to determine differences in maturation in dependence of temperature conditions.

The females were weighed on day 30 when they entered the experimental condition and thereafter at least once a week to the nearest 0.1 g on an electronic Sartorius balance. Adult females were weighed in addition on the day of parturition. Young were weighed on the day of birth and for the first 5 days of life. Thereafter they were weighed every 5 to 7 days until day 30 when they were separated from their mothers unless they were still not weaned. The experiment lasted until both females in a given enclosure had given birth to and weaned three litters or for 260 days after the females had been put into the enclosures, whichever occurred first. This is more than enough time for littering three times given the pregnancy duration of 68 days and postpartum estrus.

We determined parturition time and litter size, sex ratio and mass of individual pups at birth by daily controls. To determine the time of weaning we observed nursing events from day 20 onwards every day for 30 min. Females frequently also nurse the offspring of other females in the enclosure while nursing their own litter. Therefore, we counted nursing events by any female in the enclosure. If no suckling was observed for two subsequent days, the first day without suckling was defined as weaning age. When two females gave birth simultaneously, the last day when a given female was observed to nurse any pup was determined and then the first day thereafter was taken as time to weaning. In some cases, pups were not observed suckling on day 20 or later and these cases were recorded as weaned at an age of 20 days.

To determine body composition, we used the calibration curves established by Raffel et al. (1996). Briefly, lean body mass was measured using an EM-SCAN SA-2 small animal body composition analyser (EM-SCAN Inc., Springfield, Illinois, USA). The instrument measures total body electrical conductivity. Animals were first weighed to the nearest 0.1 g and then anaesthetized in a small container through which a mixture of 3% Halothan (Halocarbone Laboratories, South Carolina, USA) and air (mixing by Sulla 19; Drägerwerk, Lübeck) flowed at a rate of 2.0 L/min. Animals were sedated after about 4 min and had fully recovered ≤ 10 min later. Length (in cm; from tip of nose to the end of the vertebral column) was measured on the totally relaxed anaesthetized animal lying on its back with the top of its head resting flat against a measuring board. The animal was then introduced head first lying on its back on a plastic holder into the measuring chamber of the SA-2 analyser. The index of total body electrical conductivity (TOBEC#) was recorded by computer. Seven to 10 measurements in peak mode were taken at a given time (depending on the profundity of anaesthesia) and the mean of the measurements used for further analysis. In a few cases we obtained negative measures. These were excluded as they are clearly erroneous. Following the recommendation of [12] for prediction of fat free mass (FFM) we used the regression:

$${\rm{FFM}}\left( {\rm{g}} \right){\rm{ = 3}}{\rm{.192 + 2}}{\rm{.109 *}}{\left( {{\rm{TOBEC\# *length}}} \right)^{0.5}}$$

for animals ≤ 23 cm body length and for larger animals.

$${\rm{FFM}}\left( {\rm{g}} \right){\rm{ = - 65}}{\rm{.25 + 2}}{\rm{.604*}}{\left( {{\rm{TOBEC\# *length}}} \right)^{{\rm{0}}{\rm{.5}}}}{\rm{.}}$$

Absolute fat mass (AFM) was determined as the total body mass minus the fat free mass (FFM).

### Statistical analyses

Body mass development and reproductive characteristics of females were analysed using mixed effects models (lme4; lmerTest packages, [13, 14]). Female Identity and group identity were included as random effects and temperature treatment (2 level factor) was always included as main fixed effect. For the analysis of body mass development, we additionally included female age as fixed effect and for the reproductive parameters litter mass and litter size, we included the litter number as a covariate. The litter size for guinea pigs usually shows an increase after the first litter [10] rather than following a linear trend over time. Weaning was analysed including litter size as a covariate since it is known that offspring of large litters grow slower [15]. For the analysis of birth interval, only temperature treatment was included as fixed effect. For the measurements of reproductive output, i.e., litter size, time to weaning and birth interval, we additionally ran likelihood ratio tests (function “ranova”) to investigate the amount of variance explained by maternal identity.

Temperature was the sole fixed effect in the analysis of female body length at the end of the experiment. Since we only analysed one data point per female in this case, group identity was the only random effect included in the model. For the analyses of absolute fat mass (AFM) the models included female identity as random effect and temperature treatment, female fat free mass at measurement as well as their interaction as fixed effects. To analyse potential treatment effects on sex ratio, we constructed a binomial mixed effects model (1 = male, 0 = female) with treatment as fixed and maternal identity as random effect.

To analyse potential effects of the temperature treatment on the offspring, we build two models analysing the body mass development until day 30 (~ shortly after weaning). First, we restricted the analyses to the body mass development within the first three days after birth. Guinea pig pups growing up under stressful conditions tend to lose body mass shortly after birth (pers. observation) while pups growing up under benign conditions often manage to increase in body mass already within the first couple of days. A second model was built covering the full range of body mass development from birth until day 30. Both models included individual identity, mother identity and Box identity as random effects and temperature treatment, age, sex and their two-way interactions as fixed effects. Offspring AFM at day 30 was analysed including mother identity as random effect, offspring mass, sex, litter number and their two-way interactions as fixed effects.

All models assumed a Gaussian distribution. Assumptions of all models were validated visually using q-q plots and plotting of residuals versus fitted values. P-values were obtained using the Satterthwaight´s method for approximating degrees of freedom for t-statistics.

Timing of maturation of female pups that were born in cold versus warm conditions, respectively, was tested using a proportion test.

## Results

### Females

**Fig. 1:**
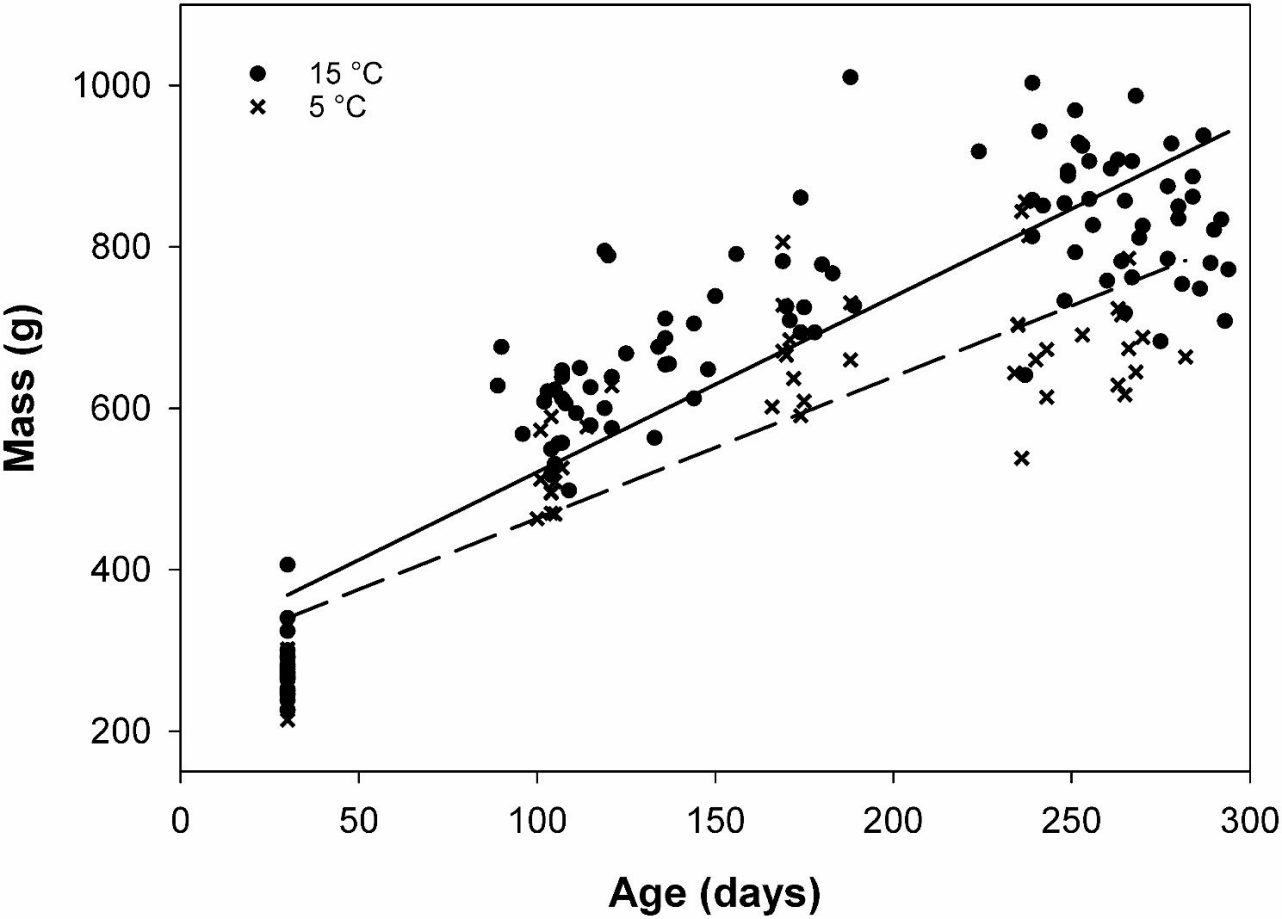
Female growth with age. Females living in a temperature of 5 °C grew slower than those in 15 °C and reached a lower final mass at the end of the experiment. All body mass measurements are of non-pregnant females

At the start of the main experiment, female mass did not differ between the two experimental groups (N = 12 females per treatment, t = 0.60, p = 0.57). Thereafter, females in the cold treatment grew slower and reached a lower body mass at 110 days of age (Fig. 1; t = 3.10, p = 0.003).

Likewise, females kept under cold conditions showed a tendency to grow less in body length (t = 2.1, p = 0.06). Temperature treatment neither affected litter mass (t = -0.65, p = 0.51), nor litter size (Table 1; t = -1.49, p = 0.14) or the interval between litters (t = 0.97, p = 0.35) across the experiment.

**Table 1:** Reproductive output of females under the two temperatures

Temperature condition	Litter #	Litter size		Litter mass (g)
5 °C	1	2.42 ± 0.51 N = 12		209.3 ± 56.0 N = 12
	2	4.27 ± 1.19 N = 11		344.1 ± 93.5 N = 10
	3	5.5 ± 1.18 N = 10		406.6 ± 83.3 N = 9
15 °C	1	2.08 ± 0.51 N = 12		228.5 ± 54.2 N = 12
	2	4.25 ± 1.60 N = 12		361.8 ± 112.0 N = 12
	3	4.18 ± 1.72 N = 11		379.0 ± 118.6 N = 11

Litter sizes and litter mass for 1st, 2nd and 3rd litters (mean ± SD) under the two temperature conditions. Not all females gave birth to a second and third litter. Litter mass for litters (N = 1) where a young had been partly eaten were excluded.

**Fig. 2:**
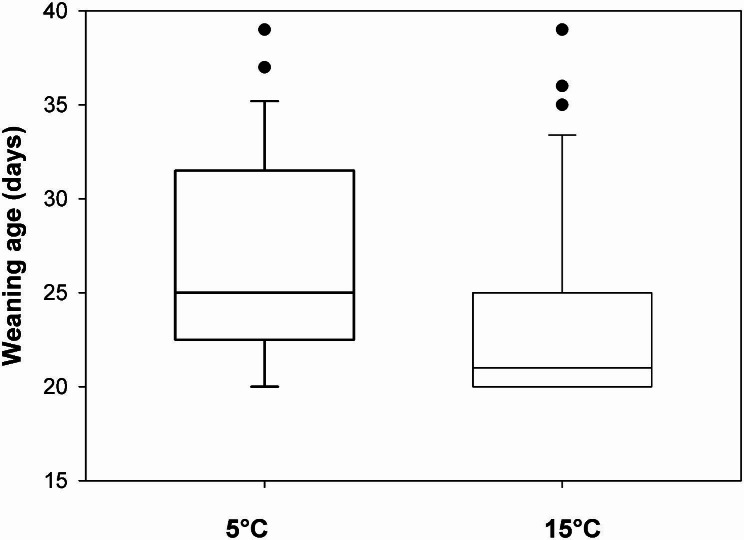
Pup weaning age. Pups growing up in a temperature of 5 °C were weaned later than pups in 15 °C (medians and quartiles; outliers). See text for statistics

With increasing female age both, litter mass (t = 4.27, p < 0.001) and litter size (t = 5.73, p < 0.001) increased independently of treatment. Females breeding under the 15 °C temperature weaned their pups earlier than females under 5 °C (Fig. 2; t = -2.1, p = 0.048). Sex ratio did not differ between treatments (z = 0.875, p = 0.38), with 48% of pups being males under 5 °C and 53% being males under 15 °C.

Maternal identity did not account for a significant variance component in any of the reproductive parameters (litter size: LRT = 0.20, p = 0.66; time to wean: LRT = 0.01, p = 1; birth interval: LRT = 0.002, p = 0.99).

**Fig. 3:**
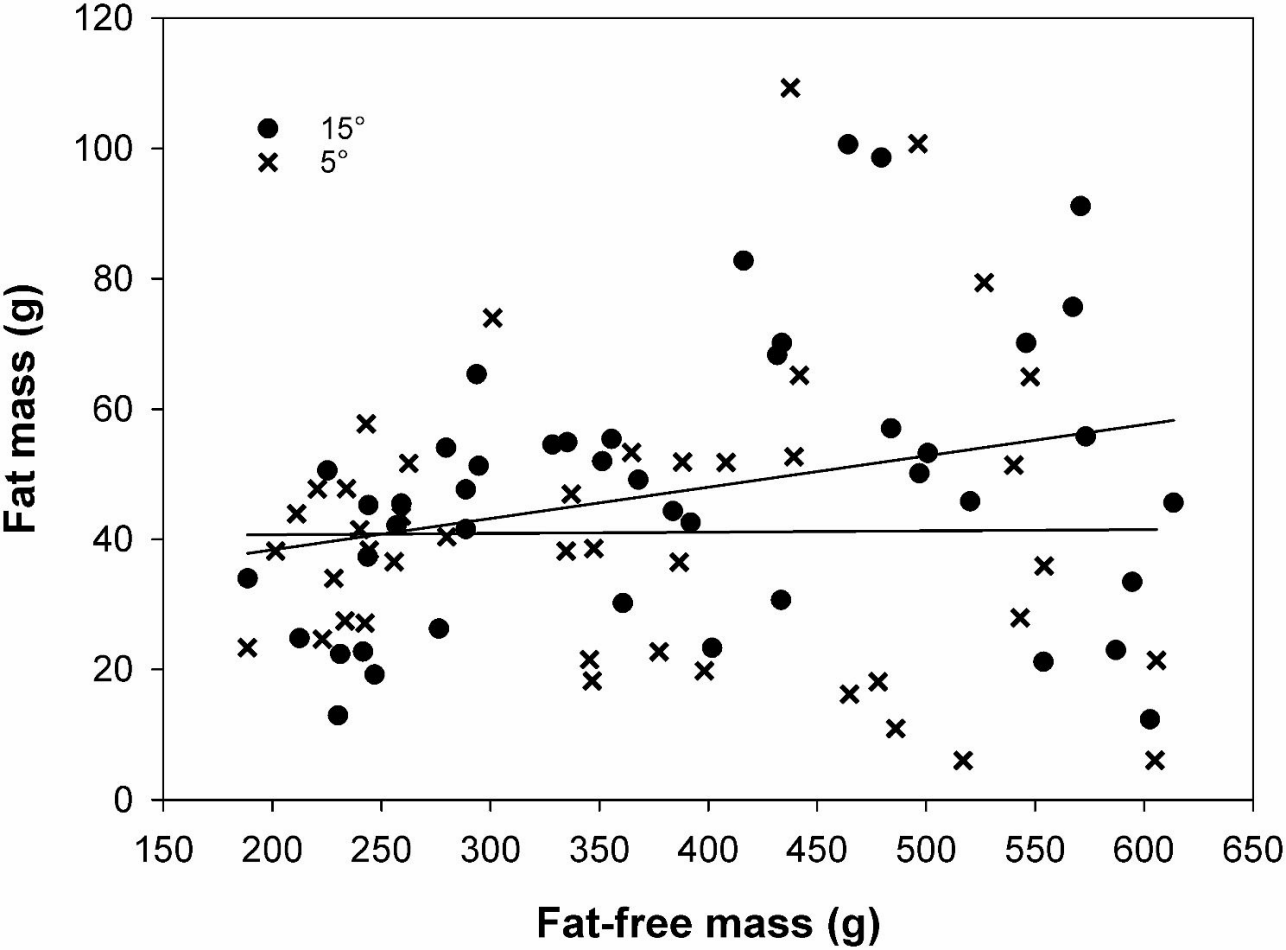
Fat storage pattern. With increasing fat-free body mass absolute fat mass increased in females living under 15 °C but remained constant in females living in a temperature of 5 °C

Over the period of the first pregnancy (day 30 to 110), female absolute fat mass (AFM) developed differently between the temperature treatments. Fat mass of females under cold conditions stayed relatively constant with increasing fat-free body mass, but for females under warmer conditions it increased with fat-free body mass (interaction temperature x AFM: t = 3.13, p < 0.003, see Fig. 3).

At the same time, increase in fat-free mass (structural growth) did not differ between the two groups (15 °C group: 291.6 ± 44.7 g; 5° group: 266.0 ± 35.9 g; interaction temperature x fat-free mass: t = -0.07, p = 0.95).

### Pup development

In total, 130 pups were born under 5 °C and 121 pups under 15 °C with pre-weaning mortality rates of 13% and 7%, respectively. In cold (5 °C) conditions, pups were born about 10 g lighter than in 15 °C conditions (mean ± SD; 81.8 ± 15.9 g instead of 92.5 ± 20.1 g; t = 3.46, p = 0.002). This effect was independent of offspring sex (interaction treatment x sex: t = -0.19, p = 0.85). During the first three days after birth, pups in cold conditions lost more body mass (t = -4.98 ± 0.49 g) than pups born into the warmer condition (-1.6 ± 0.44 g, interaction treatment x age: t = 3.67, p = 0.0003). Body mass development of pups in cold conditions was continuously slower, leading to on average 16% lower total body mass at 14 days of age (t = 7.75, p < 0.001) and 18% lower body mass at 30 days of age (Table 2; t = 15.91, p < 0.001).

**Table 2:** Characteristics of pups at 30 days of age (weaning)

Temperature condition	Litter #	Body mass (g)	Fat mass (g) AFM	% fat RFM
5 °C	1	250.7 ± 27.3 N = 25	23.6 ± 8.5 N = 25	9.5
	2	242.9 ± 31.3 N = 38	29.4 ± 12.3 N = 38	12.1
	3	242.0 ± 32.8 N = 36	28.3 ± 10.1 N = 36	13.2
15 °C	1	311.8 ± 19.2 N = 24	46.0 ± 8.3 N = 24	14.8
	2	292.3 ± 38.4 N = 47	43.7 ± 9.3 N = 47	15.0
	3	294.0 ± 60.2 N = 38	47.0 ± 20.3 N = 38	16.0

Total body mass and estimated absolute (AFM) and relative fat mass (RFM) on day 30 in surviving pups under the two temperature conditions (mean ± SD; N = number of surviving pups on day 30). These are raw means not considering litter size or sex differences. Not included are pups that could not be assigned unequivocally to a mother due to synchronous parturition of the two females in a given enclosure.

**Fig. 4:**
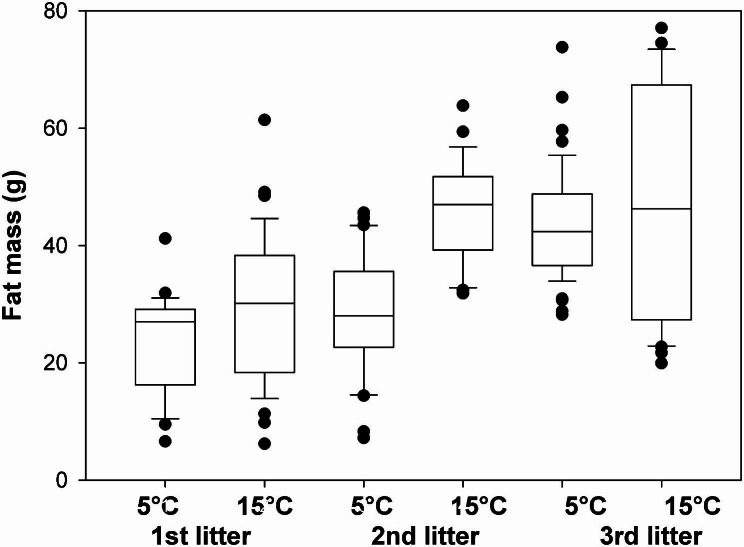
Pup fat mass. Absolute fat mass (AFM; median, interquartile range, 95% range; outliers as dots) of pups on day 30 of life as a function of their mothers’ litter number and the temperature in which they grew up

Offspring AFM on day 30 of age was significantly higher for offspring born under warmer conditions (t = 2.23, p = 0.03; Fig. 4). In addition, there was a slight increase in relative fat mass (RFM) with increasing litter number, i.e., maternal age, irrespective of treatment (t = 13.6, p < 0.001) and this increase across subsequent litters was stronger for animals under cold conditions (interaction temperature x litter number: t = 3.34, p = 0.04). Offspring sex did not influence RFM but RFM was higher in animals of higher body mass (measured as fat free mass t = 11.67, p < 0.01).

There were significant effects of mother identity on offspring developmental traits (i.e. consistent differences in maternal reproductive effort across subsequent litters), including birth mass (LRT = 9.26, p = 0.002), growth to day 30 (LRT = 14.53, p < 0.001) and RFM at day 30 (LRT = 18.55, p < 0.001) but not body mass loss during the first three days of life (LRT = 2.05, p = 0.15).

In the additional small experiment, females born in 5 °C reached sexual maturity later than females born in 20 °C: All 10 females born in 20 °C conceived until day 40 and on average gave birth on day 99.5 (± 6.8). In contrast, only 5 of the 13 females born in 5 °C produced a litter and gave birth on day 104 (± 3.7) (χ^2^ = 9.44; p < 0.01).

## Discussion

By exposing young females to cold (5 °C) or moderate (15 °C) temperatures, we aimed to test potential shifts in allocation strategies between self-maintenance (own structural growth and increase in fat tissue) and reproduction in a small mammal species. Closely related wild relatives such as *Cavia aperea* and *Cavia magna* usually face high extrinsic juvenile and adult mortality rates, leading to short live spans of usually less than a year.

### Female intra-individual allocation responses

In the cold, females grew slower indicating that they had to channel more of the available energy intake into thermoregulation as also observed in other small mammals such as rats and mice [16]. Despite access to ad libitum food they did not simply increase food intake to allocate the same proportion of resources to structural growth, fat storage and reproduction. Whether this represents an inability to increase food intake or an integrated response to the cold thermal environment cannot be decided based solely on our data. Earlier results, however, favour an integrated physiological and behavioural response to unfavourable environments. Growth rates and resting metabolic rates for example are lower in cavies experiencing autumn and winter seasons [17]. These effects can be triggered by a simulation of a decreasing photoperiod alone that is indicative of autumn [18]. In addition, animals experiencing either natural changes in season (including shifts in photoperiod and temperatures) or solely changes in photoperiod, develop different behavioural strategies. Behavioural phenotypes in autumn and winter tend to favour energy-conserving strategies with reduced locomotion activity and exploration compared to behavioural phenotypes in spring and summer [18, 19]. Similar strategies have been observed in other small mammals such as mice [16]. In addition, animals experiencing photoperiods indicative of unfavourable seasons invest more into adaptive, i.e., costly immune traits [20].

In an earlier study, body mass of female guinea pigs kept under a mild thermal regime with food and water ad libitum increased faster in non-reproducing than in reproducing females [12]. This difference in body mass resulted from more rapid fat deposition in non-reproducing females. In contrast, fat-free mass of reproducing females tended to increase at a higher rate than in non-reproducing ones [12]. Thus, female structural growth was not impaired by the onset of reproduction.

In our study, females in 5 °C stored less fat at a given body mass. It appears possible that females changed allocation from storage of white fat to higher synthesis of brown fat in reaction to the increased demand on generation of heat. Under the given ad libitum conditions, we may expect them to store more as insulation and as reserve for harsh times. However, for small mammals, insulation by fat storage is inefficient as its cost in terms of reduced mobility may soon get higher than the benefits of increased insulation. In fact, a few post-mortem dissections suggested that they built up large brown fat tissue in the neck and fore-leg axilla regions. Brown fat is highly metabolically active and by uncoupled respiration and ATP turnover generates heat that can serve to keep the brain and body warm [21]. Similar increases in brown fat tissue under cold conditions have been observed previously in house mice [22]. Thus, the ability for increased thermoregulation appears to take precedence over the storage of white fat.

Females in cold conditions grew slower but produced the same reproductive output in terms of litter size and mass. Also, the interval between litters did not differ. Overall, this indicates that investment in reproduction has high priority in this species. Indeed, a closely related wild species, the cavy (*Cavia aperea*), is exposed to high external mortality through predation by raptors and small mammalian carnivores [23]. The only clear difference in response to the thermal regime was behavioural and showed up in the time to weaning which occurred earlier under 15 °C than 5 °C. Taken together, our results indicate only a slight influence of cold temperature on the allocation decisions between self-maintenance and reproduction from the female perspective.

There was an effect of mother-ID indicating that mothers differed systematically in the amount of resources they provided to their offspring. Whether that occurred in utero or through differences in milk yield is unclear.

### Weaning and general effects on pups

Laurien-Kehnen and Trillmich [24] showed that mildly food restricted females produced pups of lower mass at birth and weaned later than females under ad libitum food quite similar to our findings of thermally challenged females. This also corresponds to an earlier finding that mothers did not react to increased food demand of pups by increasing milk transfer [25]. Similarly, Naguib et al. [26] found that pregnant females, i.e. females with higher energetic need, reacted less to pup calls. All of these results suggest that females transfer the costs of reproduction to their offspring in an inter-generational trade-off [27–29] with the result that growth and development of young are affected [30]. In other species, such effects can even reach across a number of generations as shown for house mice [31] and Syrian hamsters [32, 33].

Pups were heavier at birth and weaning under warmer conditions and stored correspondingly more fat. However, again differential build-up of brown fat may explain the difference. Surprisingly, relative fat mass (RFM) did not differ. This happened despite the fact that pups born into cold condition were initially smaller and lost substantially more mass than pups under the warmer 15 °C conditions. Pups of older mothers (producing their second and third litter) appeared to manage better. They were able to store more fat until weaning especially under cold conditions.

In conclusion, we found that females in more demanding thermal conditions matured later but did not reduce investment in structural growth. They regulated energy investment differently by reducing fat storage, increasing heat generation and slightly reducing investment in offspring birth mass and growth. How much of the saving was achieved by reduced investment in offspring body mass differed consistently among females. Under low temperature conditions pups grew less and stored less fat. Thereby mothers transfer part of the costs of living to their offspring. We hypothesize that in an environment where food resources become limiting, as is the case in a seasonal environment, these changes in allocation strategy will eventually lead to the cessation of reproduction as seen under natural conditions where the wild *Cavia magna* rarely reproduces during winter [8]. The reduced body mass of pups at birth was partly made up by more intense pup demand leading to later weaning, i.e. an extended lactation period. These effects were evident despite ad libitum food availability which either did not allow females to fully compensate their higher energy demand by increased food intake or through an intrinsic regulatory response led to a change in allocation priority.

## Data Availability

The data to this article are deposited at PUB.uni-bielefeld.de under 10.4119/unibi/2979295.
